# Transfer of amenamevir into breast milk in breastfeeding patients with recurrent herpes simplex: study protocol for a single-arm, open-label study

**DOI:** 10.3389/fped.2025.1551335

**Published:** 2025-04-10

**Authors:** Sachi Koinuma, Mikako Goto, Jumpei Saito, Atsuko Murashima, Tomoyoshi Takeshita

**Affiliations:** ^1^ The Japan Drug Information Institute in Pregnancy, National Center for Child Health and Development, Tokyo, Japan; ^2^ Department of Pharmacy, National Center for Child Health and Development, Tokyo, Japan; ^3^ Medical Affairs Department, Maruho Co., Ltd, Osaka, Japan

**Keywords:** breastfeeding, recurrent herpes simplex (RHS), amenamevir, milk transfer, relative infant dose (RID), patient initiated therapy (PIT)

## Abstract

**Background:**

Recurrent herpes simplex (RHS) is a disease caused by reactivation of the herpes simplex virus. Patients with RHS are treated with anti-herpes virus medication. Amenamevir is one such medication used for RHS in Japan and is administered as patient-initiated therapy (PIT); this involves initiation at the discretion of the patient, based on early symptoms. However, there are insufficient data on the transfer of amenamevir into breast milk among breastfeeding patients with RHS.

**Objectives:**

This study aims to assess the transfer of amenamevir into breast milk and evaluate the infant's risk of drug exposure.

**Methods:**

This study is a single-arm, open-label, interventional multicenter study. Patients who experience RHS during breastfeeding will be recruited and treated with amenamevir. The concentration of amenamevir in breast milk will be determined by liquid chromatography-mass spectrometry. The primary outcome is relative infant dose (RID) calculated by C_ave[AUC(0–24 h)]_. The secondary outcome is RID calculated by C_max_ and C_ave[AUC(0–48 h)]_.

**Discussion:**

This study will provide evidence of the transfer profile of amenamevir into breast milk during PIT. If the RID of amenamevir is less than 10%, such therapy is generally considered to be safe, and use of PIT with amenamevir may consequently lead to a new standard therapy for breastfeeding patients.

## 1 Introduction

Herpes simplex is a disease caused by primary infection or reactivation of the herpes simplex virus (HSV), which causes blisters or erosive lesions on the skin and mucous membranes (1). The disease occurs most commonly in individuals in their twenties, and the prevalence of HSV in adults is estimated at about 50% (2).

HSV is classified into HSV-1 and HSV-2, with latency in the trigeminal nerve or a dorsal root ganglion after primary infection. HSV-1 recurs mainly on the face, particularly around the lips, and HSV-2 recurs mainly on the lower body, particularly around the genitals (1). The frequency of recurrence is generally reported to be higher with HSV-2 than HSV-1, although some patients will get monthly recurrences, while others will experience this once every few years. In women, there may be a tendency for the condition to recur more easily due to the fact that progesterone secreted in the latter half of the menstrual cycle has an immunosuppressive effect (3). This is because stress caused by premenstrual syndrome disrupts the autonomic nervous system (4), and because the immune system is weakened during pregnancy, so that the mother's immune system does not recognize the fetus as a “foreign body” (5).

In Japan, the oral antiviral drugs acyclovir, valacyclovir, famciclovir, and amenamevir are clinically used for the treatment of recurrent herpes simplex (RHS). As of February 2025, acyclovir and valacyclovir are listed as safe for use during breastfeeding on a website (supervised by the US National Institutes of Health) that provides expert information on the use of drugs during breastfeeding (6). This is based on a number of recent medical studies.

In Japan, amenamevir received marketing approval in 2017 for the indication of herpes zoster (7, 8) and in February 2023 for the treatment of RHS, with approval of patient-initiated therapy (PIT), where medication can be initiated at the patient's discretion, based on early symptoms (9). Approximately 80% of patients with RHS can recognize early symptoms (tingling, itching, etc.) before skin lesions appear. PIT facilitates the suppression of viral replication from an early stage by exposing the virus to high concentrations of the medication soon after symptom onset, a strategy referred to as “early short-term treatment”. However, in Japan, only famciclovir (approved in 2019) and amenamevir are approved for PIT (9, 10).

The oral treatment of herpes simplex can be expected to work if administered as soon as possible after onset of disease symptoms. There is also a guideline stating that a sufficient clinical effect cannot be obtained if administration is not started within a day after rash onset (11). Acyclovir and valacyclovir, while safe for nursing mothers, are not approved for PIT. In some cases, it is difficult for breastfeeding women to visit to a medical institution immediately after rash onset, as bringing the infant can be challenging, and making childcare arrangements at short notice is often not a realistic option. In other cases, the patient suffers from significant stress because of the risk of infecting the infant. In this respect, PIT would be a particularly convenient treatment for breastfeeding women as they can take pre-prescribed medication as soon as they recognize early symptoms. The transfer of amenamevir into breastmilk was reported in mice (12), and we thus cannot currently recommend its use in breastfeeding women due to insufficient data regarding transfer into breast milk (13). Therefore, when patients who develop RHS during breastfeeding are taking amenamevir for PIT, treatment benefits and the benefits for the infant of continued breastfeeding must both be considered.

This study aims to evaluate the transfer of amenamevir into breast milk by administering a single dose of 1,200 mg to patients with RHS during lactation, followed by milk sampling and blood sampling, measurement of amenamevir concentration in milk and plasma, and evaluation of adverse events. This study could provide a new treatment option for patients with RHS during lactation.

## 2 Methods and analysis

### 2.1 Trial design

This study is a single-arm, open-label, multicenter interventional study. The subjects are patients who experience RHS during breastfeeding and are treated with amenamevir. The National Center for Child Health and Development (NCCHD) and four hospitals in Japan are registered as participants. This study is under the control of the principal investigator from NCCHD and sub-investigators from registered hospitals. All investigators are clinical doctors.

### 2.2 Recruitment and selection

Registrations are promoted through in-hospital public relations (posters, pamphlets), direct interviews with patients at medical institutions, and completed questionnaires. Patients with a background that could potentially be relevant are also investigated based on their past medical history. Patients who have a history of herpes simplex and are candidates for enrollment will receive an explanation of the study, together with a supplementary informed consent document during their regular check-up visits. Clinical research coordinators (CRC) from the contracting institution will support the explanations. Patients who meet all of the inclusion criteria and who do not meet any of the exclusion criteria will be eligible for enrollment. The inclusion criteria are described below.

(1) Patients who have had a diagnosis of RHS;
(2) In the past year, patients with one or more recurrences of herpes simplex;
(3) Patients who are breastfeeding within 1 year after childbirth, at the time of informed consent;
(4) Patients who are aged from 18 to 47 years at the time of informed consent;
(5) Patients with a body weight ≥40 kg and ≤70 kg at the time of informed consent;
(6) Patients who are assessed by the principal investigator or a sub-investigator as able to stop breastfeeding for 48 h after taking amenamevir; and
(7) Patients who provide voluntary, written consent to participate in the study.

Rationales for the inclusion criteria are described below.

(1–3) To target breastfeeding women with recurrent herpes simplex;
(4) Weaning of infants begins by 6 months after birth in 87.7% of cases, and 34.7% of cases are completely weaned by 12 months (14). Based on this report, we set the time limit to avoid patients dropping out due to weaning during the study;
(5) To target breastfeeding women in general; the minimum age is set taking into consideration when late teens attain reproductive age (15) and can provide informed consent on their own. The upper age limit is based on the end of the reproductive age, which is in the early 40s, taking into account the latest possible pregnancy age. Assuming the latest pregnancy age is 45 years, adding approximately one year of pregnancy and one year of breastfeeding results in an age of 47 years;
(6) Based on the general weight range for women aged 18 to 47 in Japan (16);
(7) Continuation or discontinuation of breastfeeding while taking amenamevir should be considered while taking account of the expected therapeutic benefits and the benefits of breastfeeding. However, considering the unknown actual amount of exposure through breast milk and its effects, in this study, the safety of the infants was prioritized by organizing cessation of breastfeeding for 48 h after administration. The half-life of amenamevir is approximately 7 h, and it has been confirmed that blood concentrations decrease sufficiently after 48 h in healthy adults (12).
(8) To honor ethical considerations that apply to patients.

The exclusion criteria are described below.

(1) Patients administered drugs/beverages listed in the section “Contraindications to concomitant use” or “Precautions for concomitant use” in the package insert of amenamevir, within the 14 days prior to the time of obtaining informed consent;
(2) Patients requiring dietary restrictions associated with comorbidities,;
(3) Patients with extreme dietary restriction due to weight loss;
(4) Patient with a history of amenamevir hypersensitivity;
(5) Patients with comorbidity or a history of diabetes, hepatitis B or C, tuberculosis, mastitis, or malignant tumors;
(6) Patients with a history of HIV infection or AIDS comorbidities
(7) Patients who are pregnant or have childbearing potential and intend to become pregnant during the study period; or
(8) Other patients who are considered ineligible by the principal investigator or a sub-investigator.

Rationales for the exclusion criteria are below.

(1) To avoid drug interactions and maintain an appropriate plasma drug concentration, thereby ensuring efficacy and safety;
(1–3) There is a report that indicates the impact of diet on the composition of breast milk;
(4–7) To ensure the safety of those subjects;
(8) In order for the investigator or sub-investigator to make a comprehensive decision taking into account the effects of amenamevir on blood drug levels and milk drug levels, and factors related to patients safety.

### 2.3 Screening and consent

We will use an informed consent form to explain the study to patients and obtain their voluntary written consent to participate in the study. In the unlikely event that the informed consent form (ICF) is revised due to a change in the study plan or other reasons after the patients begin participation in this study, the revised ICF must be rigorously reviewed by a certified review board. After the revised form is approved, the details of the change will be explained to patients, and adequate time will be given them to consider whether they are willing to continue their participation. We will therefore once again obtain their consent for continued participation. In addition, for patients who do not have an opportunity to visit the hospital and for whom it is difficult to obtain face-to-face re-consent, an explanatory document and ICF will be mailed, and the changes will be explained by telephone or web interview, to obtain consent.

### 2.4 Intervention

**Figure 1:**
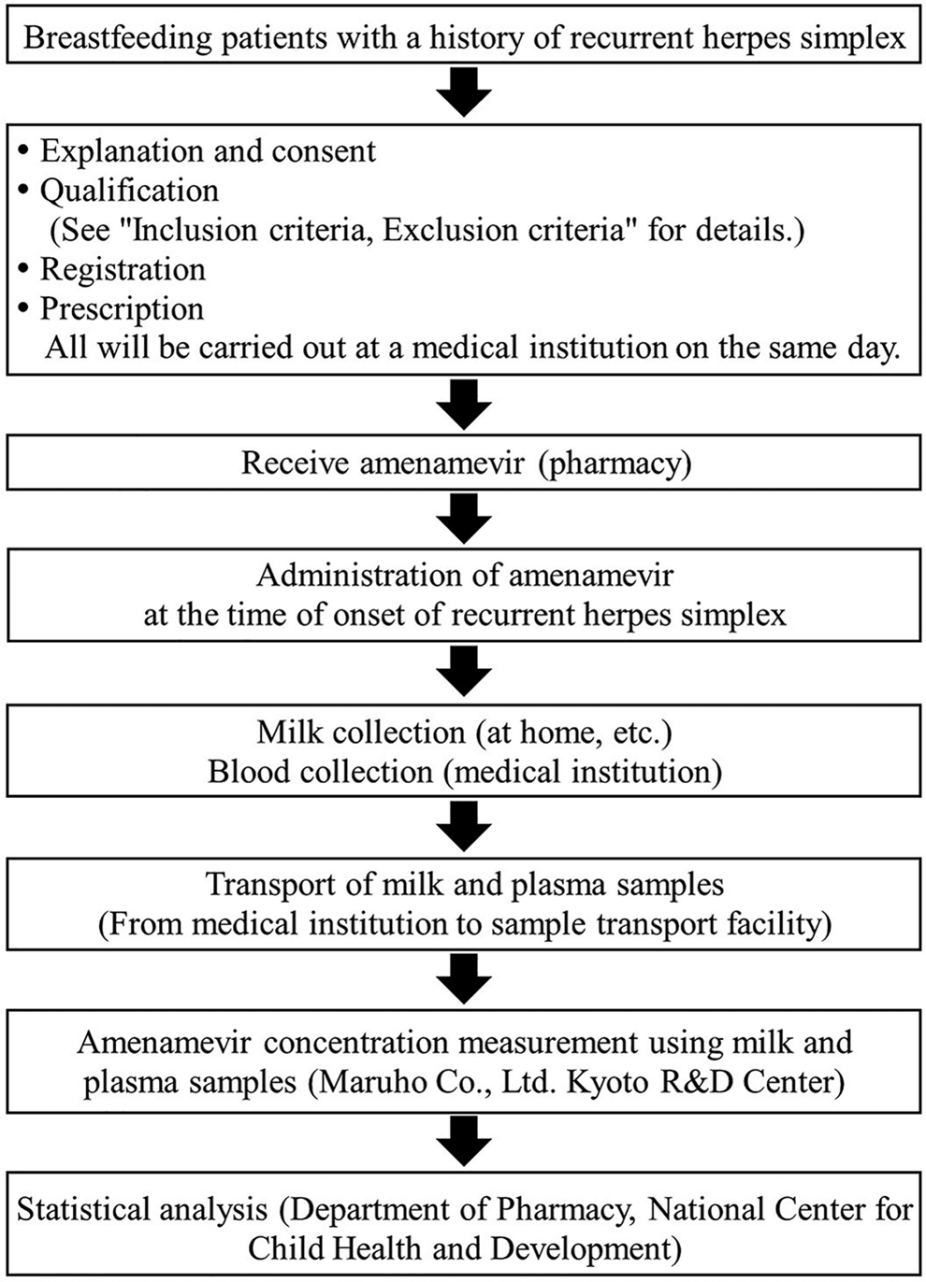
Schematic representation of this study.

Patients are prescribed 1,200 mg of amenamevir and carry the medicine with them, to take at any time. Patients take the prescribed amenamevir as PIT, taking medication at their own discretion based on early symptoms of RHS. Amenamevir should be taken after a meal, and within six hours of the earliest symptoms of RHS. Patients should avoid breastfeeding for 48 h after taking amenamevir. To avoid effects on plasma and breast milk concentrations of amenamevir due to drug interactions and other factors, the use of drugs and food items listed in the “contraindications or precautions for concomitant use” sections of the packaging insert is prohibited (Table 1). A schematic representation of this study is shown in Figure 1.

**Table 1:** Contraindications for concomitant use and precautions for coadministration.

Category	Drug name, etc.	Signs, symptoms, and measures	Mechanism and risk factors
Contraindications for concomitant use	Rifampicin (Rifadin®)	Blood concentrations of both drugs may decrease, and the effects of amenamevir and rifampicin may be reduced.	The CYP3A inductive effects of both amenamevir and rifampicin may both promote the metabolism of both drugs.
Precautions for coadministration	CYP 3 A substrate drugs (midazolam, brotizolam, nifedipine, etc.)	Blood concentrations of these drugs may decrease, and the effect of these drugs may reduce.	The CYP3A inductive effect of amenamevir may promote the metabolism of these drugs.
	Drugs that inhibit CYP3A (ritonavir, clarithromycin, etc.) Grapefruit juice	Blood concentrations of amenamevir may increase.	These drugs may inhibit the metabolism of amenamevir.
	Cyclosporine	Blood concentration of amenamevir may decrease, and the effect of amenamevir may be reduced.	The mechanism is unknown.
	Agents that induce CYP3A (rifabutin, carbamazepine, phenobarbital, etc.) Foods containing St. John's wort	Blood concentrations of these drugs may be decrease, and the effect of this and these drugs may reduce.	The CYP3A inductive effects of amenamevir and these drugs may both promote the metabolism of both drugs.
	CYP 2B6 substrate drugs (efavirenz)	Blood concentrations of amenamevir may decrease, and the effect of amenamevir may be reduced.	The CYP2B6 inductive effect of amenamevir may promote the metabolism of drugs that are substrates of CYP2B6.

CYP, cytochrome P450.

To improve medication adherence, CRC calls patients monthly to check that they received the medicine and to explain when and how to take it. If any of the following conditions are found to apply, the patient will be discontinued from participation in this study.

(1) In the event of withdrawal of consent;
(2) If it is found that the patient is ineligible based on the inclusion or exclusion criteria;
(3) If breast milk production stops;
(4) Discontinuation of the entire study; or
(5) Other than the above, when the principal investigator or a sub-investigator decides that discontinuation is warranted.

### 2.5 Outcomes

The primary outcome is the relative infant dose (RID) of amenamevir calculated by C_ave[AUC(0–24 h)]_.

The secondary outcomes are as follows:

• RID calculated by C_max_
• RID calculated by C_ave[AUC(0–48 h)]_
• Plasma concentration of amenamevir
• Frequency and rate of adverse events

RID is defined as follows:

$$\mathrm{RID}(\text{\%})=\frac{\mathrm{theoretical}\;\mathrm{infant}\;\mathrm{daily}\;\mathrm{dose}\;(\mathrm{TID})\;(\mathrm{mg}/\mathrm{kg}/\mathrm{day})}{\mathrm{maternal}\;\mathrm{medication}\;\mathrm{dose}\;(\mathrm{mg}/\mathrm{kg}/\mathrm{day})}\;\times 100$$

TID is defined as follows:

TID(mg/kg/day)=drugconcentrationinmilk(mg/ml)×infantfeedingvolume(ml/kg/day)

Drug concentration in milk uses C_ave[AUC(0–24 h)],_ C_ave[AUC(0–48 h)]_ or C_max_. The infant feeding volume is set at 150 ml/kg/day in this study (17).

### 2.6 Data collection and measurement

Detailed patient characteristics (e.g., body weight, clinical-disease type) are collected (Table 2). After administration of amenamevir, approximately 3 ml of milk is collected into individual milk storage containers at 4, 8, 24, 36, and 48 h (Table 3). The acceptable milk collection period is set at ±1, ±2, ±2, ±4, and ±4 h, respectively. The time at which a specimen of breast milk is collected is entered on both a recording sheet and the container. In addition to the required collection times mentioned above, we also request optional breast milk collection at any time within the 24 h following administration to enhance the accuracy of analysis. Patients submit breast milk samples when they visit the hospital. Blood will be drawn once in hospital (blood volume: 3 ml) within 5 to 24 h after taking the drug.

**Table 2:** Details of collection of patient characteristics.

Collection items	Details	Survey date
Date of birth, age (years)	–	• Informed consent
Body weight (kg)	–	• Informed consent • Day 0*^1^
Height (cm)	–	• Informed consent
BMI (kg/m^2^)	–	• Informed consent • Day 0*^1^
Date of birth (Month/Day) (Birth date of infant subject to breastfeeding)	–	• Informed consent
Comorbidity	–	• Informed consent
Medical history	Diabetes mellitus, hepatitis B or C, HIV infection history, AIDS, tuberculosis, mastitis, malignancy	• Informed consent
Clinical forms	Recurrent herpes labialis (facial herpes), recurrent genital herpes (herpes of the pubic colliculus and anal circumference, herpes of the gluteal region, and herpes of the lower extremities)	• Day 0*^1^
Dose of amenamevir	1,200 mg or other dose	• Day 0*^1^
Amenamevir compliance	Fasting/within 30 min after meals/taking medication more than 30 min after meals.	• Day 0*^1^

*^1^ Day 0 is the date of amenamevir treatment for early symptoms of recurrent herpes simplex.

BMI, body mass index; HIV, human immunodeficiency virus; AIDS, acquired immune deficiency syndrome.

**Table 3:** Schedule of data collection and monitoring.

Items	Registration	0 h	4 h	8 h	24 h	36 h	48 h	discontinued
Visit	○		○				○	⬤
Explanation/consent obtained	○							
Patient characteristics	○		○					
Concomitant medication	○		○				○	
Amenamevir prescription	○							
Amenamevir administration		○						
Milk collection			○*^1^	○*^2^	○*^2^	○*^3^	○*^3^	
Blood sampling			○					
Adverse event	○							
Confirmation of reasons for discontinuation								○

○: Mandatory, ⬤: optional.

*^1^ The acceptable milk collection period is set at ±1 h.

*^2^ The acceptable milk collection period is set at ±2 h.

*^3^ The acceptable milk collection period is set at ±4 h.

Participant recruitment occurred from January to November 2024, and data collection will continue until April 2025. Monitoring will be conducted throughout the entire data collection period.

For laboratory testing, 3 ml of whole blood treated with sodium heparin will be centrifuged for 5 min at 2,000 × g and the supernatant will immediately be transferred to a clean tube using a pipette, within 44 h of blood collection. Milk and plasma samples will be stored at −80℃. Amenamevir milk and plasma concentration measurements will be performed by liquid chromatography-tandem mass spectrometry (LC/MS/MS), and peak identification, calculation of peak areas and peak area ratios will be calculated. Preparation of calibration curves and calculation of quantitative values will be performed using the LC/MS/MS operation software Chromeleon 7.2.10 (Thermo Fisher Scientific Inc., Tokyo, Japan) at Maruho Co., Ltd. Kyoto R&D Center. In order to confirm the reproducibility of the quantitative values from the initial analysis, an incurred samples reanalysis (ISR) of milk and plasma samples will be performed. The ISR should be performed on a different day from the initial analysis, within a period during which sample stability is ensured. If the deviation is within ± 20% for more than two-thirds of the samples that underwent ISR, reproducibility is adjudged to be present.

### 2.7 Number of breast milk samples

For calculation of RID, in order to reduce the burden on patients as much as possible, mandatory sampling times of breast milk should be set to the minimum number required. In a study conducted on healthy adults, the t_max_ and t_1/2_ of amenamevir in plasma were 3.83 ± 0.41 h and 7.06 ± 0.29 h, respectively. Based on these data, breast milk sampling times should be set at approximately 4 h, 8 h, and 24 h, corresponding to the t_max_, t_1/2_, and disappearance as the minimum necessary time for calculating AUC_0–24 h_. Additionally, 36 h and 48 h were established as mandatory milk collection points for calculating AUC_0–48 h_.

### 2.8 The need for blood sampling

In this study, blood will be sampled 5–24 h after amenamevir administration. Amenamevir concentration in the plasma will then be measured. If RID is markedly higher or lower than expected, the patient's plasma drug concentration will be compared to data obtained from healthy adults in clinical trials (12). Verifying that the plasma concentration of amenamevir in these patients is in the range of the mean ± standard deviation plasma concentrations established for healthy adults will allow identification of major differences. The blood sampling time was set between 5 and 24 h to ensure that amenamevir can be detected in plasma without affecting breast milk sampling at 4 h, when the amenamevir concentration is assumed to be highest. If the plasma drug concentration is different from that of healthy adults, we will again need to check dietary contents, their intake status, and concomitant medications, etc., as these may affect the pharmacokinetics of amenamevir. These measures will be taken to ensure data quality is maintained.

### 2.9 Statistical methods

The analysis population will comprise the full analysis set (FAS) and per protocol set (PPS), with the primary analysis population in this study being the PPS. The FAS consists of all patients enrolled in this trial except for the following patients:

• Patients who have not received the protocol treatment
• Patients whose data are not collected after the protocol treatment starts
• Patients subsequently found to violate eligibility criteria after enrollment

The PPS consists of the population excluding the following patients from the FAS:

• Patients who deviated from the study protocol, affecting the proper assessment of the primary endpoint

Background patient information (e.g., medical history) will be described individually. In addition, the frequency of each category will be calculated and summary statistics (number of cases, mean, standard deviation, median, maximum, and minimum) will be calculated.

Pharmacokinetic parameters (AUC_0–24 h_, AUC_0–48 h_, and C_max_) will be determined for each patient using the drug concentration in milk obtained by measurement. AUC_0–24 h_ and AUC_0–48 h_ will be calculated using the linear-log trapezoidal calculation method, with C_max_ the actual measured value. TID and RID will also be calculated for each patient. The infant feeding volume is set at 150 ml/kg/day in this study (14).

Even if milk samples are not obtained at all mandatory time points in the target population, the primary and secondary endpoints will be evaluated without excluding the target population from the analysis. If it is considered possible to calculate the AUC by confirming the linearity of the elimination of amenamevir from breast milk, the primary and secondary endpoints will be evaluated. If this is impossible, all or some of the secondary endpoints will be evaluated. As the transfer of the drug into breast milk has been evaluated in few cases so far, the data obtained from all subjects will be disclosed as a data set, including the time of collection and the concentration at each time point.

Statistical analysis will be performed at the Department of Pharmacy, National Center for Child Health and Development.

Adverse events will be calculated with reference to MedDRA/J (ver. 27.1) based on the number of cases with adverse events of a given severity, study drug, and causality, as well as the percentage of the total and the number of occurrences.

### 2.10 Sample size

Sample size considerations include variability in the pharmacokinetics of the drug, study design, and variation in lactation physiology, which are determined according to the main objective; for exclusively breast milk studies, which are exploratory, a minimum of 6 to 8 subjects is considered sufficient (18). Furthermore, in clinical studies during lactation, the number of cases is often limited due to ethical and practical constraints. Recent reviews of pharmacokinetic studies on drug transfer into breast milk have not prevented research due to the difficulty in recruiting subjects and the small number of cases; indeed, studies based on data from fewer than ten cases have also been included for consideration (19). Considering that a small number of cases would result in sparse data, a study was performed to determine how many cases were needed, and the results showed that if there are samples which have two time points per case, the minimum number of cases will be 5 (20). Based on the above theory, the target number of evaluation cases will be set at 5. The frequency of recurrence varies from once every month to once every few years. Therefore, the selection criteria include a history of recurrence in the past year. In addition, during clinical trials of amenamevir, about 40% of patients did not have a recurrence during the study period. Furthermore, there is a possibility that patients will forget to take amenamevir because there will be a period between enrollment and recurrence. There is also a possibility that breast milk production may stop during the study period. Taking these into consideration, the target number of enrolled cases will be set at 20. Note that, as the average number of feeds per day for infants aged 3 months or older is generally 6 to 8, it was not deemed necessary to set the number of cases after taking into account the timing of milk sample collection.

### 2.11 Adverse event reporting and harms

For adverse events, the date of onset, severity, seriousness, predictability, outcome and outcome date, causal relationship to amenamevir, and causal relationship to the study will be assessed by the principal investigator or a sub-investigator and recorded on a case report form.

Illnesses occurring in patients will be evaluated from the time of consent until 48 h after taking amenamevir or at the time of study discontinuation. Adverse events occurring in patients will be evaluated from the time of taking amenamevir until 48 h after taking amenamevir or at the time of study discontinuation. However, if treatment for an adverse event that occurred during the study period persists even after 48 h have passed since taking amenamevir, the follow-up period will be until the completion of treatment. In principle, follow-up will be conducted until the adverse event that occurred has recovered or improved, or until the principal investigator and co-investigator determine that it is no longer necessary or possible to follow up on the adverse event that occurred. Among the adverse events that may occur in patients, information on adverse reactions accumulated from clinical trials is shown in Table 4 (21). The half-life of amenamevir is approximately 7 h, and it has been confirmed that blood concentrations decrease sufficiently after 48 h (12). Since patients will stop breastfeeding for 48 h after taking the medication, amenamevir will not be transferred to the infant, and therefore adverse events on the infant will not be evaluated. However, if, for various reasons, an illness occurs in a breastfed child within 48 h after taking amenamevir, the same measures as above will be taken, but the record will be in the illness report for the drug. The period for collecting information on the child's illnesses, etc. will be from the time of breastfeeding within 48 h after taking amenamevir to 48 h later.

**Table 4:** Adverse reactions.

Category	≥1%	<1%	Incidence unknown
Hypersensitivity		Drug eruption (including erythema, eczema, rash)	Urticaria, itching
Psychiatric and nervous system		Headache, heaviness of head, dizziness, numbness	Dysgeusia, somnolence
Renal	NAG increased, α1- microglobulin increased	BUN increased, protein present in urine	Blood creatinine increased
Hematologic		FDP increased, basophil count increased, eosinophil count increased, lymphocyte count increased, red blood cell count decreased, white blood cell count decreased, white blood cell count increased, platelet count increased, neutropenia, monocyte count increased	Hematocrit decreased, hemoglobin decreased, anemia
Hepatic		ALP increased, hepatic function abnormal, liver function test abnormal, hepatic enzyme increased, ALT increased, direct bilirubin increased, blood bilirubin increased	γ-GTP increased, AST increased
Gastrointestinal		Diarrhea, feces soft, gastritis, nausea, abdominal discomfort. abdominal distension, abdominal pain, vomiting, paresthesia oral, stomatitis	Constipation, flatus, thirst, decreased appetite
Cardiovascular		QT prolonged, hypertension, blood pressure increased, ST elevated, palpitations	Heart rate increased
Others		Blood uric acid increased, urine sugar positive, periodontitis, tooth abscess, blood cholesterol increased, amylase increased, blood chloride decreased, blood potassium increased, malaise, chills, pyrexia, pain in extremity, dyspnea, visual impairment, color blindness, photophobia	Erythema multiforme*^1^, edema, nasopharyngitis, protein total decreased

*^1^ Clinically significant adverse reactions.

NAG, β-N-acetyl-D-glucosaminidase; BUN, blood urea nitrogen; FDP, fibrin degradation products; ALP, alkaline phosphatase; ALT, alanine aminotransferase; γ-GTP, γ-Glutamyl transpeptidase; AST, aspartate aminotransferase.

## 3 Discussion

The results of this study will provide essential data on the transfer profile of amenamevir in breast milk during PIT. Although there have been reports of acyclovir and valacyclovir being transferred to breast milk during 5-day treatment (22, 23), there have been no reports of drugs administered by PIT being transferred into breast milk. For the assessment of infant safety, an RID level of 10% or less is generally considered to be safe (24, 25). If the RID of amenamevir is identified, this could provide a benchmark for deciding whether to administer amenamevir to patients. Therefore, these findings are anticipated to contribute significantly to the development of safe and effective treatment options for breastfeeding women with RHS.

Finally, PIT is a rational approach for patients, particularly breastfeeding mothers, enabling timely and effective treatment while balancing infant safety. By addressing the practical challenges faced by these patients, such as the difficulty of accessing immediate medical care, this study aligns with patient-centered care principles. As this study targets a small number of breastfeeding women (5 to 6 cases), it will not be possible to evaluate inter-individual variability in amenamevir concentrations in breast milk. In addition, as collections may be sparse, it may not be possible to calculate accurate values for amenamevir transfer into breast milk. Furthermore, as the participants in the study will not breastfeed within 48 h after taking amenamevir, it is impossible to evaluate the influence of the drug on infants when acquired by breastfeeding.
